# A Self-Powered and Autonomous Fringing Field Capacitive Sensor Integrated into a Micro Sprinkler Spinner to Measure Soil Water Content

**DOI:** 10.3390/s17030575

**Published:** 2017-03-12

**Authors:** Eduardo Ferreira da Costa, Nestor E. de Oliveira, Flávio J. O. Morais, Pedro Carvalhaes-Dias, Luis Fernando C. Duarte, Andreu Cabot, J. A. Siqueira Dias

**Affiliations:** 1 Department of Semiconductors, Instruments and Photonics, School of Electrical and Computer Engineering, University of Campinas, Campinas, SP 13083-820, Brazil; eduardoc@cpqd.com.br (E.F.d.C.); neo@fee.unicamp.br (N.E.d.O.); flaviojom@tupa.unesp.br (F.J.O.M.); pcdias@utfpr.edu.br (P.C.-D.); lfduarte@utfpr.edu.br (L.F.C.D.); 2 Department of Electrical Engineering, Paraná Federal University of Technology—UTFPR, Cornélio Procópio, PR 86300-000, Brazil; 3 Faculty of Science and Engineering, São Paulo State University Júlio de Mesquita, Tupã, SP 17602-496, Brazil; 4 Catalonia Institute for Energy Research (IREC), Jardins de les Dones de Negre 1, Barcelona 08930, Spain; acabot@irec.cat; 5 Institució Catalana de Recerca i Estudis Avançats (ICREA), Pg. Lluís Companys 23, Barcelona 08010, Spain

**Keywords:** autonomous sensors, soil water content sensors, capacitive soil water content sensor, energy harvesting, micro sprinkler spinner generator, ultra-low-power circuits

## Abstract

We present here the design and fabrication of a self-powered and autonomous fringing field capacitive sensor to measure soil water content. The sensor is manufactured using a conventional printed circuit board and includes a porous ceramic. To read the sensor, we use a circuit that includes a 10 kHz triangle wave generator, an AC amplifier, a precision rectifier and a microcontroller. In terms of performance, the sensor’s capacitance (measured in a laboratory prototype) increases up to 5% when the volumetric water content of the porous ceramic changed from 3% to 36%, resulting in a sensitivity of S=15.5 pF per unity change. Repeatability tests for capacitance measurement showed that the $\theta_{v}$ sensor’s root mean square error is 0.13%. The average current consumption of the system (sensor and signal conditioning circuit) is less than 1.5 μA, which demonstrates its suitability for being powered by energy harvesting systems. We developed a complete irrigation control system that integrates the sensor, an energy harvesting module composed of a microgenerator installed on the top of a micro sprinkler spinner, and a DC/DC converter circuit that charges a 1 F supercapacitor. The energy harvesting module operates only when the micro sprinkler spinner is irrigating the soil, and the supercapacitor is fully charged to 5 V in about 3 h during the first irrigation. After the first irrigation, with the supercap fully charged, the system can operate powered only by the supercapacitor for approximately 23 days, without any energy being harvested.

## 1. Introduction

Precision agriculture practices demand accurate and autonomous sensors [1,2]. However, due to the soil space variability [3], these sensors need to be deployed in large numbers, especially in large crop fields, which requires them to be economic and self-powered, i.e., able to harvest their energy from the environment. In particular, soil moisture sensors are essential to implement irrigation management.

Capacitance sensors are usually low-cost, easy to install, and provide a reliable estimation of the soil moisture volumetric content by measuring the dielectric permittivity of the soil, which depends on the amount of water [4,5,6,7,8]. The energy required to measure soil water content using capacitive sensors is typically three orders of magnitude lower than with sensors based on the heat dissipation principle [9,10,11,12,13,14], making them an excellent choice to be used with cost-effective low-power energy harvesting modules.

Although photovoltaics and thermoelectrics are commonly used in most energy harvesting systems, in irrigated agriculture the use of hydraulic energy is particularly convenient and cost effective. Hydraulic energy can be conveniently collected from the rotating movement of the micro sprinklers spinners used to homogeneously irrigate the field.

The goal of this work is to develop an autonomous and self-powered system to measure soil moisture and control irrigation. With this goal in mind, we coupled a capacitive sensor to measure soil water content with an interrogation circuit that includes an ultra-low power microcontroller. The whole system was powered by an energy harvesting module which uses a micro sprinkler spinner, and it was able to perform automatic irrigation management through a water latching valve.

## 2. System Design and Fabrication

### 2.1. Operation of the Sensors in a Crop Field

**Figure 1:**
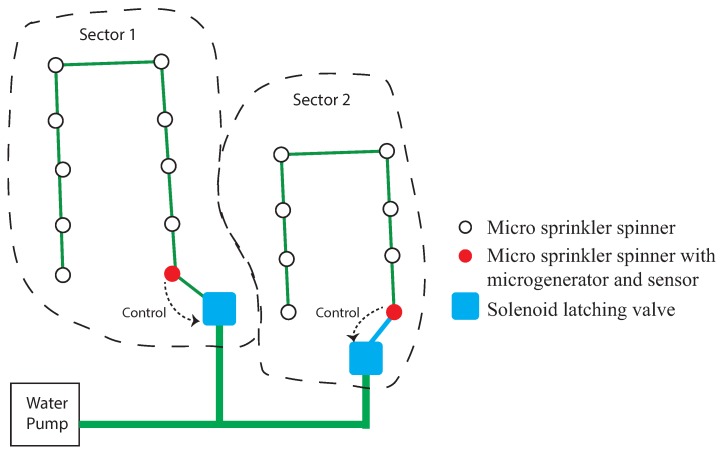
A diagram of the installation of the sensors in a crop field.

A diagram of the sensor location within the micro sprinkler spinner network in a crop field is shown in Figure 1. In this scheme, the irrigation of two parcels of soil is controlled by two sensors and two valves. The operation of the proposed irrigation management system is as follows.

The system is installed with the valves open. When the irrigation pump is turned on for the first time, the water flows through the micro sprinklers spinner, and a DC voltage is generated by the microgenerator located on the first micro sprinkler spinner (in the direction of the water flow), next to a valve.

The generated voltage is detected by the energy harvesting circuit, and the microcontroller makes a soil moisture reading. If the soil moisture is below the desired level, the microcontroller leaves the valve open, and all the micro sprinklers keep irrigating that parcel of soil. If the soil moisture is at the desired level, the microcontroller keeps the valve open for about 3 min (time that the energy harvesting system requires to charge its supercapacitors), and then sends a pulse to the latching valve, closing it.

Since it is necessary to start the system with the valve open, after a few hours (after the irrigation pump was turned off), the microcontroller sends another pulse to open the valve, preparing the system for the next operation of the irrigation system.

### 2.2. Design and Fabrication of the Capacitive Sensor Based on a Porous Ceramic to Measure Water Content

The capacitive sensor was fabricated using a conventional PCB (Printed Circuit Board), as proposed by Dean et al. [15], with a 45 mm × 25 mm × 0.5 mm FR-4 substrate material with Cu foil on both sides. The electrodes are made of interdigitated copper tracks (94 fingers, with a length L=16 mm, width W=200 μm and space between fingers S=200 μm), which were patterned on the top side of the PCB. The W/S relationship which maximizes the value of capacitance per unity area has been calculated in [16,17,18] as W=S. The value of *L* was determined simply by the porous ceramic size, because if L>>S, as in most cases, the fingers can be considered infinite [18].

As in the work presented by Dean et al. [15], a continuous Cu backside plane was left on the bottom of the PCB. This Cu backplane left on the PCB is important to minimize the change in the capacitance of the sensor due to the influence of the water with backside fringing fields. It is important to observe that the sensing volume of this sensor is very small, because only the soil which is above the electrodes will effectively influence the sensor’s capacitance.

Instead of using a solder mask to provide electrical insulation [15], the PCB was coated with a thin PDMS layer (Polydimethylsiloxane) on both sides using a spin coater, creating a very thin layer (15 μm after baking) on the top of the electrodes. This thinner layer of PDMS maximizes the sensitivity of the sensor in relation to the dielectric material that is placed over it. The sensor practically does not respond to changes in dielectrics which are placed at distances greater than W+S from the sensor’s surface [19].

However, the accuracy of the sensor’s readings when inserted directly in the soil depends on the good contact between the electrodes and the soil, and inaccurate and false results can be obtained if there are air gaps between the soil surface and the sensor [7]. To minimize these problems, we used a porous ceramic placed on the sensor’s electrodes.

**Figure 2:**
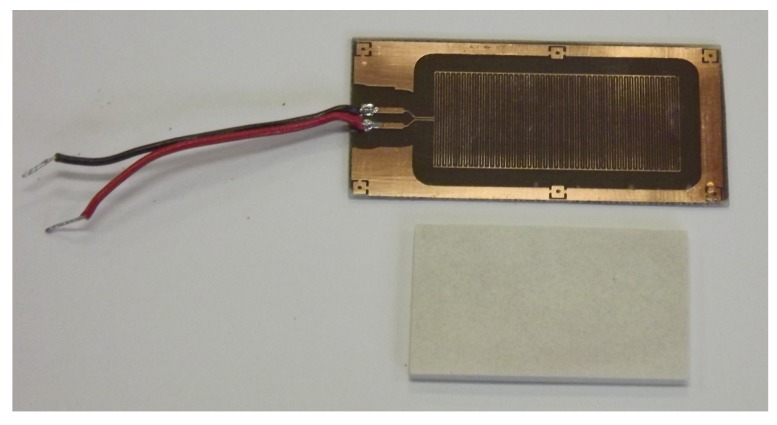
Photograph of the PCB with interdigitated electrodes (**top**) and a porous ceramic plate (**bottom**).

A porous ceramic plate (39 mm × 20 mm) with a thickness of 2 mm was placed on the top side of the PCB, completely covering the electrodes. The porous ceramic plate was firmly pressed to the PCB coated with PDMS (applying a 100 g weight evenly distributed over the ceramic plate) and its sides were sealed with a RTV (Room Temperature Vulcanization) silicone, bonding the porous ceramic plate to the PCB. A photograph of the PCB with the interdigitated electrodes and the porous ceramic is shown in Figure 2.

**Figure 3:**
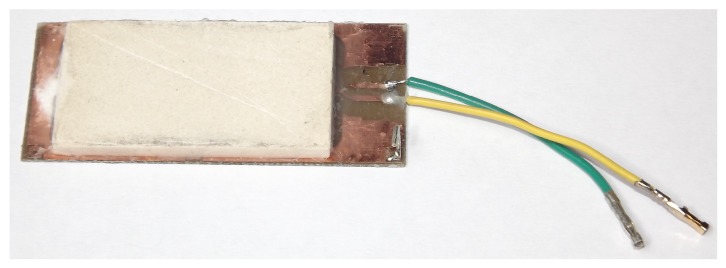
Photograph of the fabricated sensor.

Since the electrodes are totally covered by the porous ceramic plate, the changes in the capacitance will be caused only by the amount of water absorbed into the porous ceramic. A photograph of the sensor with the porous ceramic is presented in Figure 3.

The use of a porous ceramic plate, that absorbs the soil water by capillarity until equilibrium is reached, represents a clear improvement when compared with the sensor presented in [15]. The use of a ceramic plate allows for more accurate measurements of the soil water potential by minimizing the problems of uneven soil contact that can result in inconsistent soil moisture measurements [20]. The contact problem that may exist in coarse sandy soils can be solved by surrounding the porous ceramic with a slurry of fine silica [9]. Although there is a delay time required for hydraulic equilibrium of the water in the soil and ceramic, which depends on both the magnitude of the water potential gradient and the hydraulic conductivity, this delay is in the order of tens of minutes.

### 2.3. The Signal Conditioning Circuit

**Figure 4:**
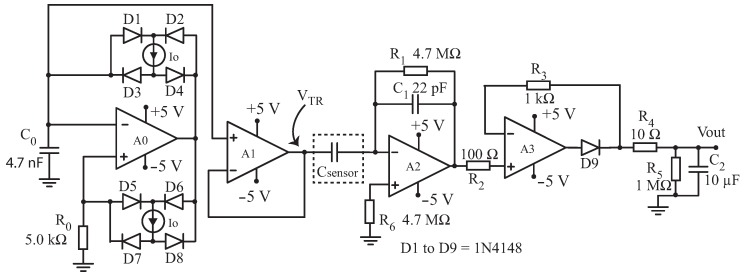
Schematic diagram of the signal conditioning circuit.

The sensor is read by a capacitance-to-voltage conversion circuit [21,22,23,24]. The developed circuit, presented in Figure 4, is based on the work presented by [21], and provides an output voltage which is proportional to the capacitance $C_{sensor}$. We tested the circuit with both sine and triangular wave signals in the input, and the measured performance was the same in both cases. Therefore, we used the simple and accurate triangle wave generator, which was designed using a precision, low temperature drift current source REF200 from Texas Instruments, Dallas, TX, USA. The triangle wave is obtained from a relaxation oscillator which charges and discharges capacitor $C_{0}$ with the current furnished by the $I_{0}=100$ μA (REF 200) current source. The comparison points are determined by the voltage drop on resistor $R_{0}$. Neglecting the input current of the op-amp $A_{0}$, the voltage drop on $R_{0}$ is given by $V_{R0}=\pm R_{0}I_{0}$, where $I_{0}=100$ μA is obtained from the same REF200 current source.

The value of $C_{0}=4.7$ nF is calculated to give a frequency of oscillation f≈10 kHz in the triangle wave, and with a 500 ${\mathrm{mV}}_{p}$ amplitude, op-amps $A_{0},A_{1},A_{2}$ and $A_{3}$ are the low-cost TL051AC from Texas Instruments, Dallas, TX, USA.

The triangle wave available on $C_{0}$ is coupled, with a unity gain buffer $A_{1}$, to an inverting amplifier, composed of $A_{2},R_{1},C_{sensor}$ and $C_{1}$. Since $R_{1}$ is very high (4.7 MΩ) and both $C_{1}$ and $C_{sensor}$ are in the range of a few tens of pF, the AC gain in op-amp $A_{2}$ is approximately given by $G_{2}\approx C_{sensor}/C_{1}$. The last stage of the signal conditioning circuit is a precision rectifier ($A_{3},R_{3},D_{9}$) and a first-order RC filter ($R_{4},R_{5},C_{2}$). After the steady state is reached, the circuit output $V_{out}$ will be a positive DC voltage proportional to $C_{sensor}$:

(1) $$V_{out}\approx \frac{C_{sensor}}{C_{1}}\cdot 500\;\mathrm{mV}$$

**Figure 5:**
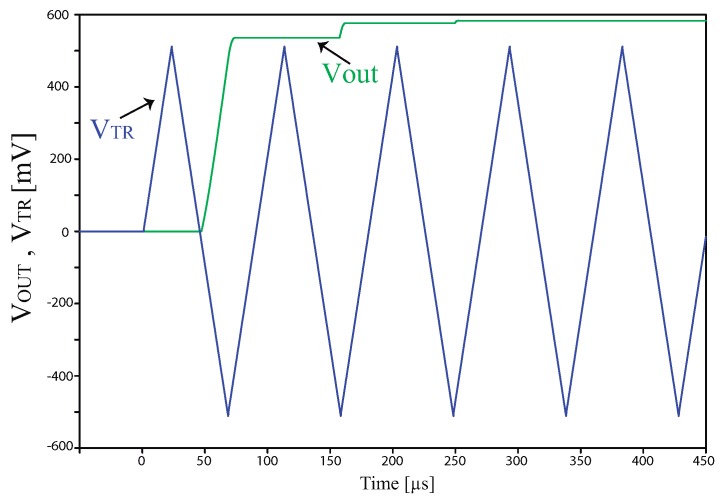
$V_{out}$ and $V_{TR}$ as a function of the time, for $C_{sensor}=30$ pF.

The value of $V_{out}$ is sent to a 12 bit A/D converter, available in the ultra low-power microcontroller (MSP430FR4959 from Texas Instruments, Dallas, TX, USA), that consumes approximately only 0.35 μA in low-power mode. The microcontroller also controls the energy harvesting circuit and the latching solenoid valve which manages the irrigation in the parcel of the field where the sensor is installed. It is important to notice that the signal conditioning circuit needs to operate only for 6 ms, since the voltage $V_{out}$ reaches the steady state in approximately 300 μs and the A/D converter requires only 5 ms to complete 10 conversions and calculate the average value of these 10 conversions. A PSPICE (Orcad) simulation of $V_{out}$ as a function of the time, for $C_{sensor}=30$ pF, is shown in Figure 5.

### 2.4. The Energy Harvesting System

**Figure 6:**
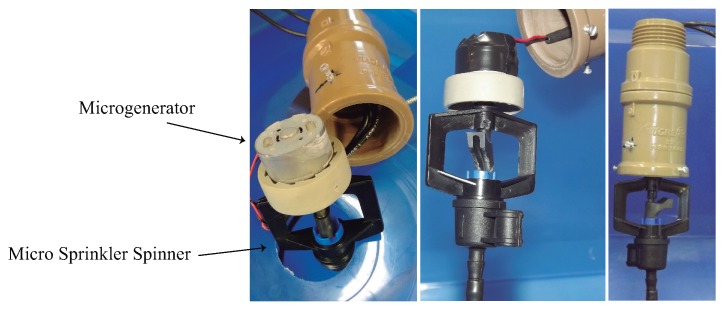
Photograph of the DC microgenerator adapted on the top of a conventional micro sprinkler spinner.

The energy harvesting system is based on a DC micro generator (ZSFD-WH6, from BDTF-MOTOR, Shenzhen China Merchant Energy Saving Technology Co., Ltd., Guangdong, China) attached to the top of an irrigation micro sprinkler spinner (Figure 6), which feeds its output voltage into a DC/DC converter that charges a 1 F supercapacitor .

**Figure 7:**
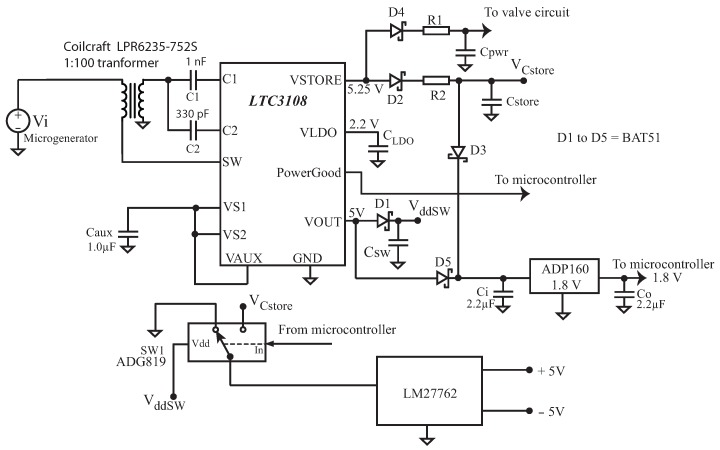
Schematic of the energy harvesting circuit.

Since the generator’s output is typically in the order of only 250 mV DC when loaded, a circuit based on the low-voltage step-up DC/DC converter LTC3108 (from Linear Technology, Milpitas, CA, USA) was used. The simplified schematic diagram of the energy harvesting circuit is presented in Figure 7. As suggested by [25], the LTC3108 internal circuits are isolated from the capacitors $C_{store}$ and $C_{pwr}$ with diodes $D_{2},D_{4}$ and $D_{5}$, since the quiescent current of the LTC3108 is too high (up to 9.5 μA) and would discharge a 1 F supercapacitor in less than four days.

The power-up sequence of the energy harvesting circuit is as follows: when the water starts flowing through the micro sprinkler spinner, a voltage $V_{i}\approx 250$ mV appears in the microgenerator, and the capacitor $C_{aux}$, connected to the $V_{aux}$ pin of the LTC3108, begins to charge. As soon as $V_{aux}$ reaches 2.5 V, the pin $V_{out}$ (which is programmed to reach $V_{out}=5.0$ V) begins to charge $C_{sw}$ through diode $D_{1}$. The capacitor $C_{i}$ also charges with the rise of $V_{out}$, and as soon as it reaches 1.8 V, the output of the low dropout voltage regulator (LDO) ADP160 (from Analog Devices, Norwood, MA, USA) powers the microcontroller with 1.8 V.

When $V_{o}$ has charged to within 7.5% of its regulated voltage (5 V in our case), the PGD output (that is connected to the microcontroller) will go high and the microcontroller detects this transition. At this point, capacitor $C_{sw}$ will be charged with $V_{Csw}\approx$ 5.0 V, and this voltage will be used to power the single pole single throw switch $S_{w1}$ (ADG819, from Analog Devices, Norwood, MA, USA).

After $V_{o}$ goes up to 5 V, a current is sourced by the $V_{store}$ pin of the LTC3108 and capacitors $C_{pwr}$ and $C_{store}$ begin to charge. If this operation is the first operation of the circuit (power-up from zero), the microcontroller will monitor (every 10 min) the voltages at $C_{store}$ and $C_{prw}$, until they charge up to their maximum values (considering the voltage drops in diodes $D_{2}$ and $D_{4}$, $V_{Cstore}\approx 4.95$ V and $V_{Cprw}\approx 5$ V).

Since $R_{2}\approx 2R_{1}$ and $C_{pwr}=20,000$ μF and $C_{store}=1$ F, $C_{pwr}$ will charge much faster than $C_{store}$. After the microcontroller detects that $C_{store}$ has reached more than $V_{Cstore}=4.9$ V (this requires about three hours of continuous operation of the sprinkler microgenerator), the microcontroller sends a pulse to the valve control circuit, closing it and preparing the system for the first controlled irrigation, which will take place probably in the next day.

In the next operations of the circuit (after power-up has been completed), when the water starts flowing through the micro sprinkler spinner and the voltage $V_{i}\approx 250$ mV appears in the microgenerator, as soon as $V_{o}$ has charged to within 7.5% of its regulated voltage and the microcontroller detects the transition on the PGD pin, the system is left running with the valve open for 3 min, which is enough to charge $C_{pwr}$ from 0 to 5 V (just in case the leakage current of the conventional 20,000 μF electrolytic capacitor is too high and discharges it during the last 24 h). After 3 min, the microcontroller turns on switch SW1, and $V_{Cstore}$ powers the charge pump LM27762 (Texas Instruments, Dallas, TX, USA), which, in turn, supplies +5 V and −5 V to the signal conditioning circuit.

Next, the microcontroller starts an interrogation of the capacitive sensor, to read the soil moisture. If the soil moisture is within the desired level, the microcontroller sends a pulse to the valve circuit, closing the valve and interrupting the irrigation in all micro sprinklers spinners in that parcel of soil. If the soil needs to be irrigated, the valve is left open for the amount of time previously stored in a look-up table stored in the microcontroller, which indicates the amount of irrigation time required, as a function of the present soil moisture.

Finally, after the irrigation pump is turned off, the microcontroller sends a pulse to the valve circuit, opening the valve and preparing it for the next irrigation operation.

## 3. Results and Discussion

### 3.1. Signal Conditioning Circuit

**Figure 8:**
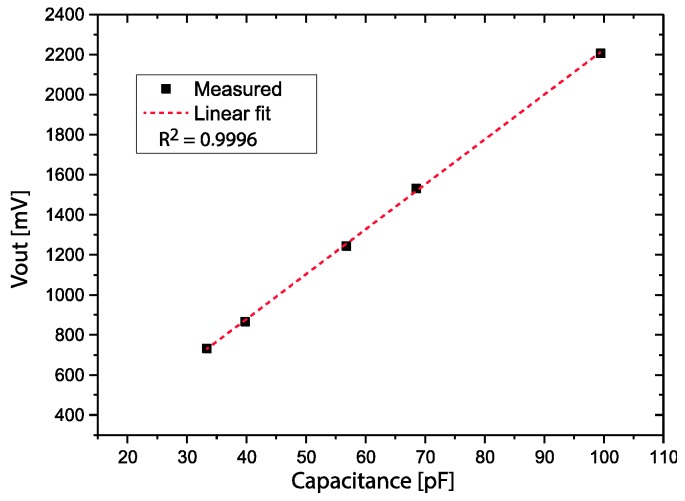
$V_{out}$ as a function of the capacitance.

The signal conditioning circuit was characterized and calibrated using ceramic capacitors, and the plot of $V_{out}$ as a function of the capacitance is shown in Figure 8. The ceramic capacitors used to calibrate the signal conditioning circuit were initially measured using a GenRad 1659 RLC Digibridge. The maximum non-linearity deviation was calculated from the measured points and a linear fit was 0.43%.

In a system installed in the field, the signal conditioning circuit must operate in a wide temperature range, typically 0 ${}^{\circ }$C to 70 ${}^{\circ }$C. This variation of temperature affects the performance of the signal conditioning circuit.

Although the REF200 current source presents a ±0.5% deviation from the 100 μA nominal current, this error in the amplitude of the triangle generator can be compensated for during initial calibration of the sensor. The same principle applies for the off-set voltage ($V_{os}$) and the input bias current ($I_{bias}$) of all op-amps of the signal conditioning circuit. However, these parameters present a drift with temperature which will cause errors in the measurement of capacitance. We will calculate the error of each component, assuming that the circuit is calibrated at 35 ${}^{\circ }$C.

The REF200 current source has a very low temperature coefficient TC=±25 ${\mathrm{ppm}/}^{\circ }$C and if $R_{0}$ is a metal foil 5 kΩ±0.01% precision resistor with a very low TC (±1 ppm/ ${}^{\circ }$C) (USR 2-0808 from Riedon, Alhambra, CA, USA), the nominal amplitude of the triangle wave will present a maximum error of ±0.45 mV (0.09%).

For the used op-amps (TL051AC, Texas Instrments, Dallas, TX, USA), the errors due to the typical values of the off-set voltage and input off-set current are very low (approximately 1.1 mV). However, a worst case analysis must be done because the maximum drift of the off-set voltage with temperature is very high ($dV_{os}/dT=25$ $\mu V{/}^{\circ }$C), and the worst case total contribution of the off-set voltage of op-amps $A_{2}$ and $A_{3}$ to the error will be ±1.7 mV. Assuming that R1 and R6 are well matched, the input off-set current of op-amp A2 contributes to a non-zero output voltage with zero input because of the voltage drop across resistors R1 and R6 (both with 4.7 MΩ). Although at 35 ${}^{\circ }$C all this error can be nullified during calibration, at 70 ${}^{\circ }$C the input off-set current can increase to a maximum value of 1 nA (worst case) and cannot be compensated for. Therefore, the drift of the input off-set current in $A_{2}$ contributes to an error of 4.7 mV.

In our prototype, designed and built only to prove the concept of the system in the laboratory, the errors would be high, approximately 6.4 mV, if the circuit reaches 70 ${}^{\circ }$C. In our sensor, which, as we will see in the next sections, has a maximum sensitivity $\theta_{v}/V_{out}=0.58$ %/mV in the region of $22.5\%<\theta_{v}<40\%$, this represents an error of 3.7% in $\theta_{v}$, and this is obviously unacceptable if we need to measure soil moisture with a 1% precision.

However, in a commercial system, this issue can be easily solved with the use of better op-amps, such as the LTC1047 from Linear Technology, Norwood, MA, USA.The LTC1047 presents (maximum values): $V_{os}=10$ μV, $dV_{os}/dT=0.05$ $\mu V{/}^{\circ }$ and $I_{bias}=30$ pA. An signal conditioning circuit built with this op-amp would present a maximum error of 144 μV, or only 0.05% in $\theta_{v}$ in the critical region of $22.5\%<\theta_{v}<40\%$. The slew-rate (SR) of high precision and low-power op-amps is usually low, and the LTC1047 (which draws only 60 μA from the power supply) has SR=0.2 V/μs. If the rise time of the output voltage in op-amp $A_{0}$ is set to 2% of the rise time of the triangle wave $V_{TR}$, the operation frequency of the oscillator circuit is limited to 20 kHz, and this is the reason we used f=10 kHz.

### 3.2. Sensor

The porous material used to make the sensor is a commercial porous ceramic used for the fabrication of soil moisture sensors, designed to operate with water matric potentials between −2000 kPa and −10 kPa [26]. The average pore size is 11 microns and the porosity is approximately 39%.

After the porous ceramic is glued to the PCB, a special saturation procedure was used to avoid the presence of entrapped air in the pores. The sensor was soaked for twelve hours in water (at atmospheric pressure), followed by an additional one hour under at a pressure of approximately 70 kPa. Then the sensor was put on a scale (with a 0.1 g resolution) and left to dry in an ambient atmosphere with controlled temperature (25±1 ${}^{\circ }$C). On the basis of the dimensions and the observed weight change from dry to saturated, the volumetric water content of the porous ceramic was calculated as $\theta_{v}=36$%. While the water was evaporating, i.e., at different values of $\theta_{v}$, several measurements of the capacitance were taken.

**Figure 9:**
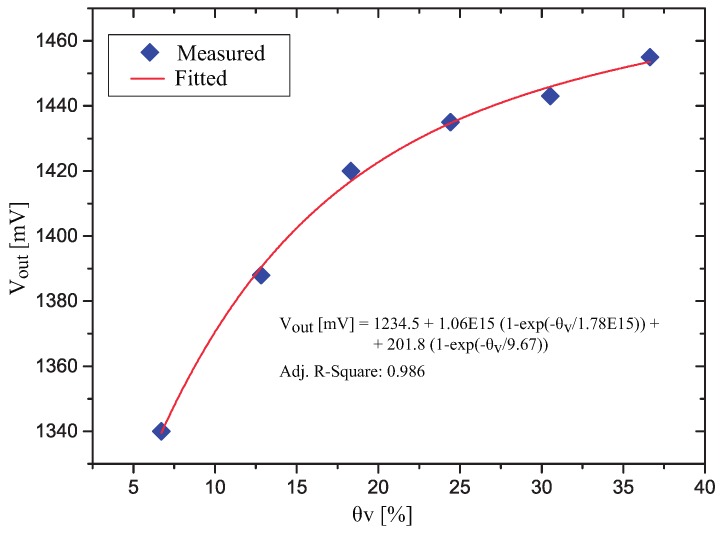
$V_{out}$ as a function of $\theta_{v}$ in the porous ceramic.

A plot of the measured voltage $V_{out}$ as a function of $\theta_{v}$ in the porous ceramic is presented in Figure 9. Observing Figure 9, we see that the variation in the output of the signal conditioning circuit was 115 mV (corresponding to a capacitance variation from 60.56 pF to 65.69 pF (ΔC=5.13 pF)). The measure points were fitted by a sum of two exponentials (Equation (2)) and the maximum deviation of the measured points from the fitted line is 2.83 mV.

(2) $$V_{out}=1234.5+1.06\times 10^{15}\left[1-\exp\left(-\frac{\theta_{v}}{1.78\times 10^{15}}\right)\right]+201.8\left[1-\exp\left(-\frac{\theta_{v}}{9.67}\right)\right]\;\left[\mathrm{mV}\right]$$

The total capacitance of a multilayer interdigitated capacitor with distinct dielectric materials is represented by series connected capacitors, one capacitor representing each pair of dielectric materials which are in contact [16,17]. Our sensor is composed of three dielectrics (FR-4 substrate, PDMS film and porous ceramic), and one of these capacitors (with FR-4 and PDMS dielectrics) is constant, while the other capacitor (with PDMS and porous ceramic dielectric) changes its capacitance value with the amount of water absorbed into the ceramics.

**Figure 10:**
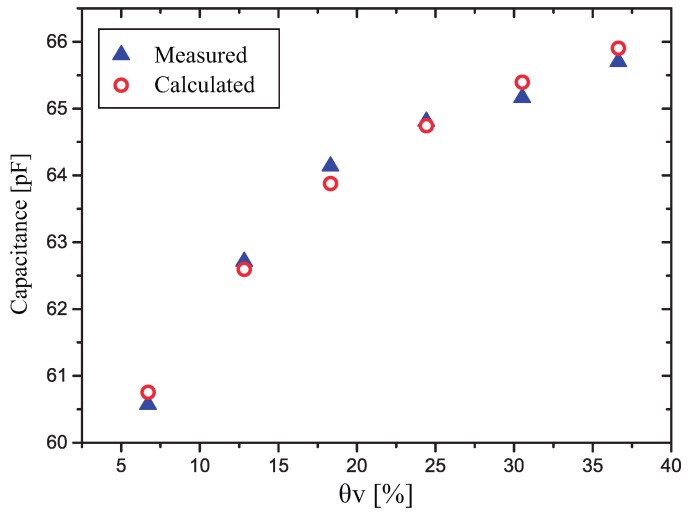
Comparison of the measured capacitance with the calculated capacitance (as a function of $\theta_{v}$) of a sensor with two series capacitors, one with a constant value (75 pF) and one varying linearly with $\theta_{v}$, from 460 pF to 1126 pF.

To show that the measured capacitance points in our sensor can be represented by a series connection of a fixed capacitor and a variable capacitor, we present in Figure 10 the total capacitance of a fixed capacitor (75 pF) in series with a variable capacitor (460 to 1126 pF) compared to the measured capacitance in our sensor. It is important to notice that the values of these capacitors were found using a numerical method, intended only to fit the measured points, and were not calculated by using the conformal mapping technique presented in [17].

The operation of porous ceramic soil water matric potential sensors is well known, and depends basically only on the characteristics of the porous ceramics. However, since each type of soil has a different water retention curve [27], a calibration procedure with the porous ceramic into the soil is required to obtain the $\theta_{v}$ of the soil [9].

The output variable in the soil moisture sensor is $V_{out}$ and, to perform the irrigation management, it is important to establish a simple relationship between $\theta_{v}$ and $V_{out}$, so that the microcontroller can calculate the value of $\theta_{v}$ from the digitized measured values of $V_{out}$. In Figure 10, a plot of $\theta_{v}$ is presented as a function of $V_{out}$, obtained from the same measured points presented in Figure 9.

**Figure 11:**
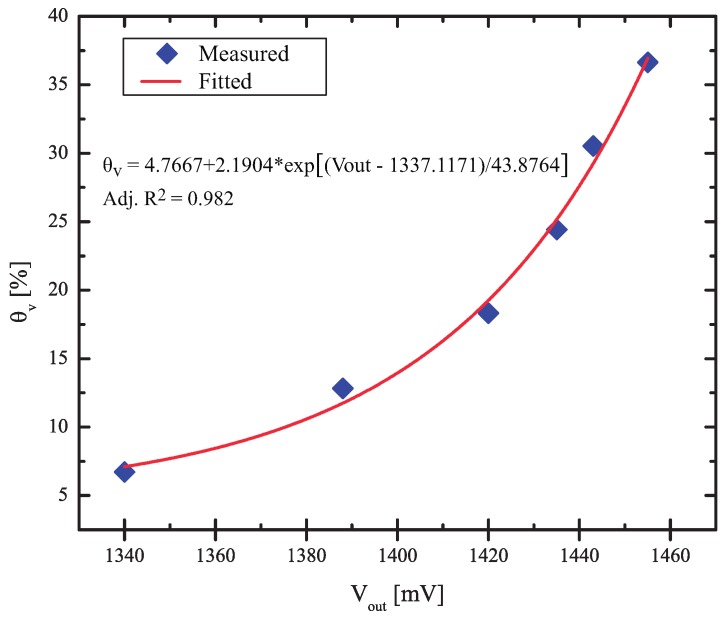
$\theta_{v}$ in the porous ceramic, as a function of $V_{out}$.

The points were fitted by a simple exponential curve, given by:

(3) $$\theta_{v}=4.7667+2.1904\cdot exp\left[\frac{V_{out}-1337.1171}{43.8674}\right]\;\left[\%\right]$$

and this is the equation used in the microcontroller to estimate the value of $\theta_{v}$ from the measured values of $V_{out}$. In the sensor that we fabricated, the porous ceramic saturated with $\theta_{v}=36$ and, although there would be small variations between porous ceramics in a production line, we assumed that $\theta_{v}$ will not reach values greater than 40%. Thus, the full-scale value of the sensor is assumed to be 40%, and we extrapolated the curve of Figure 11 to this value, obtaining a full-scale voltage of 1460 mV. Now, all errors refer to the full-scale sensor: $\theta_{v}=40\%$ and $V_{out}=1460$ mV.

**Figure 12:**
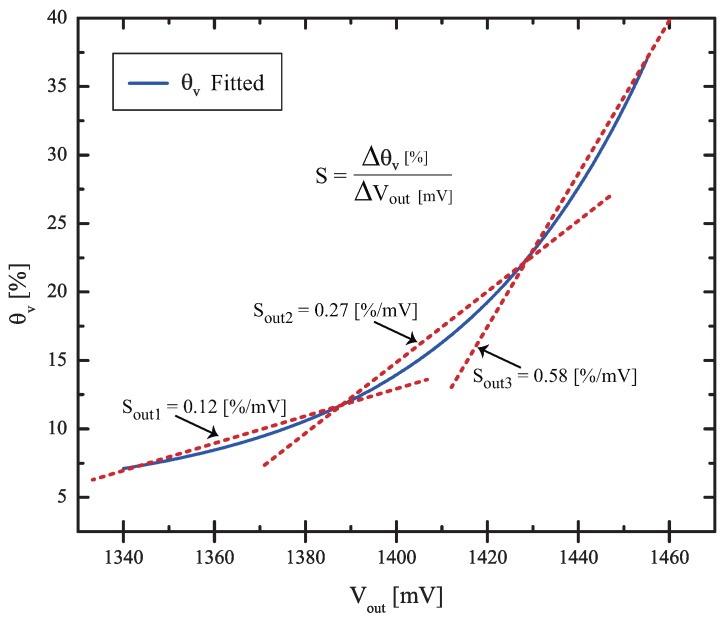
Linear approximation of the sensitivity $S_{out}=\Delta \theta_{v}/\Delta V_{out}$ in three different regions.

Although the sensor is clearly non-linear, the usual parameter of sensitivity *S* of the soil moisture sensor (defined as the variation of the sensed parameter (capacitance) per unity change ($m^{3}\;m^{-3}$) , was calculated as S=15.2 pF per unity change using the extreme points of the $\theta_{v}$ range. However, a very interesting parameter is the sensitivity of $\theta_{v}$ with respect to the output variable $V_{out}$. Due to the non-linearity, we calculated the sensitivity of the sensor as shown in Figure 12, making a linear approximation and calculating the slope $S=\theta_{v}/V_{out}$ in three different regions. The smaller sensitivity is found to be $S_{out1}=0.12$ %/mV, the higher sensitivity is $S_{out3}=0.58$ %/mV, and the medium range sensitivity is $S_{out2}=0.27$ %/mV.

The higher sensitivity region ($S_{out3}=0.58$ %/mV) is important to determine the maximum errors allowed for the signal conditioning circuit, and to check if the sensor can measure soil water content with the required precision. Since a measurement of soil water content with 1% precision is usually required [28], using the value of $S_{out3}=0.58$ %/mV, we conclude that the total voltage errors in $V_{out}$ must be smaller than 1.72 mV, including the errors due to the 12 bits A/D converter. This can be easily achieved by the system with the LTC1047 op-amp (error of 0.14 mV, as calculated in Section 3.1) and the 12 bit A/D converter with a 2.0 V internal reference (which presents a maximum error of ±0.48 mV), resulting in a reading with a maximum total error of only 0.35% in $\theta_{v}$.

We conducted tests with the sensor wetted ($\theta_{v}=18\%$) using high conductivity tap water ($\sigma =32.2\;\mathrm{mS}\cdot m^{-1}$) and rain water (σ=5.7 $\mathrm{mS}\cdot m^{-1}$), and we found that, in this conductivity range, the electric conductivity of the water does not influence the performance of the capacitive sensor, since the measured capacitance in both cases was $C_{sensor}=64.12\pm 0.013$ pF. However, just to verify the influence of the water’s conductivity in the capacitance sensor, we wetted the sensor ($\theta_{v}=18\%$) with microelectronics grade deionized water (σ=5.5 $\mu S\cdot m^{-1}$). The measured capacitance ($C_{sensor}=62.0$ pF) presented a 3% difference from the measured values with tap and rain water. This represents an almost 50% variation in the total range of capacitance change (ΔC=5.13 pF), showing that the conductivity of the water affects the sensor, and its calibration must be done using water in the adequate conductivity range.

To conduct a repeatability test, the firmware of the microcontroller was altered to make six repeated measurements of capacitance (one measurement every 5 s) at five different values of $\theta_{v}$. It is important to notice that during the 30 s while the measurements are made, the sensor is not disturbed and the water content in the ceramic is constant (within 0.1 g, the resolution of the scale).

Table 1 shows the results of the repeatability test for capacitance measurement. The highest value of the standard deviation was found to be 0.54 mV, four orders of magnitude smaller than the mean value 1400.72 mV, measured at $\theta_{v}=14.6\%$. The maximum measured peak-to-peak deviation of $V_{out}$ from the mean value also occurred for $\theta_{v}=14.6\%$, and is 1.71 mV. Regarding the $\theta_{v}$ variable, the root mean square error (RMSE) of the sensor was found to be 0.13%, one order of magnitude lower than the 1% precision usually required for soil moisture measurements.

### 3.3. Energy Harvesting

To test the microgenerator adapted to the micro sprinkler spinner, we used a Shurflo 2088-592-054 water pump (Shurflo, Cypress, CA, USA), set to supply first a water pressure of 200 kPa and then 400 kPa. We measured the output of the microgenerator without load, and observed that the output voltage of the generator was $V_{gen}=0.7$ V at 200 kPa and $V_{gen}=0.8$ V at 400 kPa. Then the microgenerator was loaded by the energy harvesting circuit presented in Figure 7, and the measured output voltage dropped to $V_{gen}=250$ mV when the water pressure was 400 kPa.

To test the energy harvesting system, and particularly to be able to measure the power-up of the system with an oscilloscope, the values of $C_{store}$ (designed to be 1 F) and $C_{sw}$ (designed to be 0.1 F) were reduced to 1000 μF. The test started with all capacitors totally discharged and the microgenerator with 0 V in its output. Next, the water pump was powered on; the microgenerator started to operate, and with a four-channel oscilloscope we measured the voltages at $C_{store}$, $C_{sw}$, $C_{pwr}$ and at the output of the ADP160 LDO (1.8 V).

**Figure 13:**
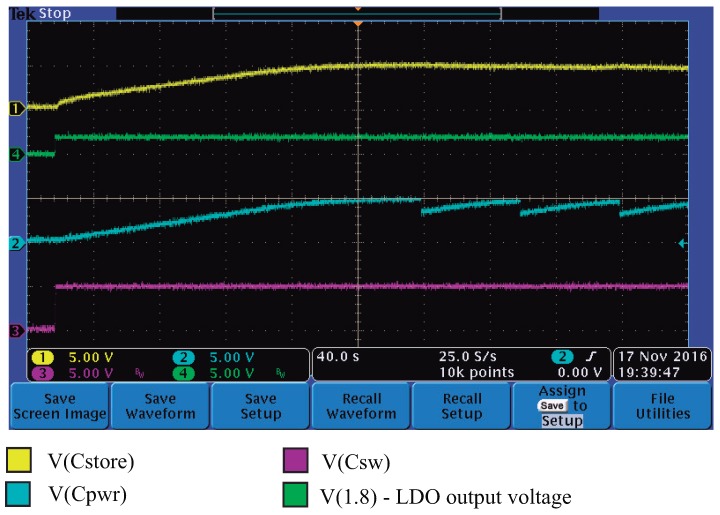
Power-up sequence in the energy harvesting circuit.

In Figure 13, we present the measured voltages as a function of the time. It can be observed that the power-up sequence starts with the LDO going up to 1.8 V (turning on the microcontroller), followed by the charge of $C_{sw}$ up to 5 V. In Figure 13, it is difficult to observe that the output of the LDO (1.8 V) occurs before the charge of $C_{sw}$ to 5 V since the oscilloscope time scale was set to 40 s/division in order to allow a complete charging cycle of $C_{store}$ and $C_{pwr}$.

After $C_{sw}$ is charged, the capacitors $C_{store}$ and $C_{pwr}$ start to charge and reach their maximum voltage approximately 100 s after the microgenerator was turned on. It is important to notice that while the microgenerator is on, the LDO is powered by $V_{out}$, since the highest voltage at the wired-or connection of D3 and D5 is $V_{out}$. However, as soon as the microgenerator is turned-off, capacitor $C_{i}$ is discharged by the LDO and its load (the microcontroller), and as soon as the voltage of $V_{out}$ drops below the voltage available in $C_{store}$, the LDO is powered by $C_{store}$.

From Figure 13, we can see that at approximately 200 s, the microcontroller sends a pulse to the valve circuit, which discharges $C_{pwr}$ very fast from 5 V to approximately 4 V (the current in the latching solenoid valve is 500 mA, applied for 50 ms). In the same Figure 13, we can see that it takes about 50 s for $C_{pwr}$ to charge again. During this test, the microcontroller was programmed to send pulses to the valve every 50 s, opening and closing the valve, as can be observed in the $C_{pwr}$ curve.

The efficiency of the LTC3108 depends only on its input voltage, and is given by its internal switching circuitry. From the LTC3108 data-sheet, we see that for a 250 mV input, the efficiency (with respect to $V_{out}$) is fixed and is only 10%. However, the efficiency with respect the $V_{store}$ pin is much lower (in this condition the current is limited to approximately 0.57 mA [29]). The energy furnished to the $V_{store}$ pin output, calculated using the energy stored in $C_{store}$ and $C_{pwr}$, is only 0.27 mW, approximately 1.2% of the 22 mW energy available at the LTC3108 input. Concerning the microgenerator, the input resistance of the LTC 3108 is determined by its internal circuitry, and with Vin = 250 mV, from the LTC3108 data-sheet we see that we have $R_{in}\approx 2.8$ Ω. Thus, the efficiency of the power that the generator (which has $R_{out}\approx 13$ Ω) with its load (LTC3108 with $R_{in}\approx 2.8$ Ω) can be easily calculated as η=17.7%.

The current consumption of the LDO ($I_{LDO}\approx 1$ μA) and the microcontroller ($I_{mcu}\approx 0.35$ μA) creates a load current to $C_{store}$ of only 1.3 μA. The average current consumption of the sensor and the signal conditioning circuit is negligible and does not contribute to the discharge of $C_{store}$ because it operates with a current of 6 mA for only 6 ms in one day. Therefore, when discharged by a current of 1.3 μA, a supercapacitor $C_{store}=1$ F charged with 4.9 V will take about 23.5 days before it reaches 1.81 V and the microcontroller stops operating. Thus, a fully charged $C_{store}=1$ F is able to power the whole system for about 23.5 days without energy harvesting from the microgenerator.

This results in a system operation which is practically perpetual, because the irrigation pump must be turned on almost everyday to make a soil moisture measurement to check if irrigation is required in any part of the field, and this will recharge the capacitors for 3 min.

Even if there are more than 24 days in a row without irrigation and, consequently, no energy was harvested during this period, the microcontroller will stop operating since it will drain all the energy stored in the supercap. However, this does not pose a problem to the system. It will power-up correctly during the next irrigation, and the power-on reset of the microcontroller will put it in the “first irrigation” mode, charging all capacitors needed for energy store, so that the system will be able to operate correctly again for 23 days in a row without energy harvesting.

## 4. Conclusions

An ultra low-power irrigation control system, based on a porous ceramic capacitive soil water sensor fabricated with PCB technology was developed. The soil water sensor and its signal conditioning circuit were powered by an energy harvesting module based on a micro sprinkler spinner. The energy harvesting system uses the movement of the micro sprinkler spinners, and with a DC/DC converter, generates all the energy required by the total system. The voltage at the output of the microgenerator installed at the top of the micro sprinkler spinners is very low (typically 250 mV), and it was collected using a low-power DC/DC converter based on the LTC3108 integrated circuit.

The energy harvesting system stores its energy in one supercapacitor that, when fully charged, can maintain the irrigation system (sensor, signal conditioning circuit and valve) operating for 23.5 days without being charged by the microgenerator. A very simple, low-cost and low-power capacitance-to-voltage conversion circuit was developed, based only on off-the-shelf electronics components. The signal conditioning circuit provides a capacitance-to-voltage conversion, and when tested with ceramic capacitors measured with an RLC bridge, showed a maximum non-linearity of 0.43% in a wide range of operation (from 30 pF to 100 pF).

Prototype sensors were fabricated with conventional PCB technology. Laboratory tests with volumetric water content in the ceramic changing from $\theta_{v}=3$% to $\theta_{v}=36$% resulted in a capacitance variation of ΔC=5.13 pF and a sensitivity of S=15.5 pF per unity change. Regarding the sensitivity of $\theta_{v}/V_{out}$, we found that in the critical region of the sensor (with $22.5\%<\theta_{v}<40\%$) we have $S_{out3}=0.58$ %/mV and with a 12 bit A/D converter, the sensor can read soil water content with a resolution of 0.28% in $\theta_{v}$. The maximum error in this critical region is ±0.38%.

A repeatability test showed that the largest value of the standard deviation for the output variable $V_{out}$ was found to be 0.54 mV, four orders of magnitude smaller than the mean ($\bar{V_{out}}=1400.72$ mV, at $\theta_{v}=14.6$%). The maximum measured peak-to-peak deviation of $V_{out}$ from the mean value is 1.71 mV. The $\theta_{v}$ root mean square error (RMSE) of the sensor was found to be 0.13%, one order of magnitude lower than the 1% precision usually required for soil moisture measurements.

The complete system was submitted to a functional test in the laboratory. The pump of the microsprinkler (which was installed inside a plastic tank) was turned on manually (as in a conventional irrigation system). The sensor was not installed into the ground, but left in open air. The porous ceramic was wetted manually, simulating an irrigated soil; the capacitance value was acquired by the A/D converter of the microcontroller and the opening/closing of the valve was controlled by the system, which performed as expected.

## Figures and Tables

**Table 1:** Measured $V_{out}$ [mV] in the repeatability test.

$V_{\mathrm{out}}$			$\theta_{v}\left[\%\right]$		
	14.6%	18.4%	20.7%	25.7%	28.0%
Meas. # 1	1401.37	1416.99	1425.78	1437.50	1441.89
Meas. # 2	1399.90	1416.50	1425.29	1436.52	1441.89
Meas. # 3	1399.90	1416.99	1424.80	1437.01	1441.89
Meas. # 4	1400.88	1417.97	1424.80	1437.99	1441.89
Meas. # 5	1400.88	1417.48	1424.80	1437.50	1441.41
Meas. # 6	1401.37	1416.99	1424.32	1438.48	1441.89
Mean Value	1400.72	1417.15	1424.97	1437.50	1441.81
Standard Deviation (*σ*)	0.54	0.38	0.38	0.49	0.14
